# Preface to Swiss National Center of Competence in Research: Molecular Ultrafast Science and Technology

**DOI:** 10.1063/1.5018454

**Published:** 2018-01-08

**Authors:** Thomas Feurer, Ursula Keller

**Affiliations:** 1 Institute of Applied Physics, University of Bern, Sidlerstrasse 5, 3012 Bern, Switzerland; 2 Department of Physics, ETH Zurich, CH-8093 Zurich, Switzerland

National Centers of Competence in Research (NCCR) are funded by the Swiss National Science Foundation to strengthen research in areas of strategic importance for the future of Swiss science, business, and society. NCCRs promote research of the highest quality with a strong emphasis on innovative and interdisciplinary approaches. NCCRs also aim to establish collaborations between academic and non-academic public and private partners, contribute to knowledge and technology transfer, support talented doctoral students and postdocs, and improve the career prospects of women in sciences.

The NCCR MUST (Molecular Ultrafast Science and Technology) was launched in 2010 and currently brings together 19 Swiss research groups working in Ultrafast Science across the fields of physics and chemistry. Besides its scientific activities, MUST has a rich program in advancement of women, technology transfer, and education outreach. The Women Professors Forum (eth-wpf.ch) is starting to become an establishment at Swiss universities, and the book “A Journey into Time in Powers of Ten” (Editors Anna Garry and Thomas Feurer) has helped to promote ultrafast science at schools and public events. MUST has also been instrumental in establishing several start-up companies.

The collection of articles in this special issue is intended to summarize and review the research carried out within the NCCR MUST and also to give perspectives for future research directions. For centuries, scientists have used theoretical modeling and imaging techniques to understand things invisible to the naked eye and thus advance our knowledge. New theoretical and experimental tools foster new science and new science requires new tools. No matter which perspective one takes, there has been, and always will be, a strong interplay between theoretical, technological, and scientific advancements. Today's requirements on theory and imaging tools have increased well beyond the known, and new frontiers in physics, chemistry, and biochemistry demand the ability to theoretically model and visualize events evolving on ultrafast time and nanoscopic length scales. As a result, MUST scientists are constantly pushing the limits of ultrafast theoretical and experimental visualization tools in pursuit of understanding electronic, atomic, and molecular dynamics and their correlations in space and time.

A solid theoretical foundation relies on an adequate modelling of atoms, molecules, and solids interacting with electromagnetic fields and the dynamics induced by the latter. Within MUST, this is pursued on different levels of complexity and quantum, semi-classical, and classical models are used to describe ultrafast dynamics. Ultrafast phenomena are often accompanied by the occurrence of non-adiabatic effects which, from a theoretical perspective, requires us to go beyond the time-independent Schrödinger equation and the Born-Oppenheimer approximation and to identify ways to solve the full time-dependent electronic and nuclear quantum problem in a highly non-equilibrium regime. Within MUST, non-adiabatic processes appear, for example, in tunnel ionization of atoms in strong laser fields, radiation-less relaxation through conical intersections, or coupling of vibrational modes.

A continued effort of several groups is to understand the implications of short time scale dynamics on long time processes. This includes vibrationally induced dissociation of small molecules in the gas phase or vibrational and internal energy redistribution through conformational dynamics but also the dynamics of proteins over many time scales. The systems investigated highlight that a comprehensive understanding of how structure, flexibility, energetics, and dynamics contribute to functional dynamics requires a concerted effort of experimental and computational studies.

A promising new experimental tool to study and control structural dynamics originates in the dramatic developments of intense pulsed THz sources. Since many dipole-active modes in solids, as well as molecules, lie in this frequency range, there is a tremendous potential to use them to study linear and nonlinear dynamics in such systems.

A large fraction of MUST activities is devoted to the understanding of charge transfer processes, which are ubiquitous in nature and play a fundamental role in many areas of chemistry, physics, biology, and materials science. More than 60 years after the seminal work of R. A. Marcus, charge transfer is still an active research field often revitalized by the ability to resolve ever faster temporal processes, even down to the attosecond time scales. Charge transfer occurs when an electron is forced to leave an atom; it occurs within molecules, between molecular centers and ligands, or along hydrogen-bonded molecular chains; and it is at the heart of most natural and artificial light harvesting processes.

For example, in light driven ionization, we have seen tremendous advances in our ability to measure the dynamics of photo-induced ionization in various systems in the gas, liquid or solid phase. MUST addresses fundamental questions such as: how long does it take to remove an electron from an atom, molecule or solid, or how many electrons are emitted in a given unit of time? But even before ionization occurs, laser fields are often strong enough to become part of the electronic potential and sometimes even dominate the Coulomb contribution. This manipulation of atomic potentials and of the associated states and bands finds fascinating applications in gases and solids, both in the bulk and at the surface and has important implications on electron dynamics.

A highly relevant topic in the context of charge transfer is electron transfer and subsequent charge separation across donor-acceptor heterojunctions, for example, in dye-sensitized solar cells, conjugated polymer- and small molecule-based organic photovoltaics or high-efficiency lead halide perovskites. It is particularly important to unravel the dynamics of photo-induced electron transfer, carrier trapping and association, energy transfer, and relaxation.

Surfaces and interfaces often exhibit a chemical activity that is different from the bulk properties. Decisive properties like charge transfer, molecular dipole, and sticking coefficient for molecular adsorption are solely determined by the local coordination of the adsorption sites and, therefore, by the structural arrangement of molecules and surface atoms. Due to the high surface sensitivity of electron-based methods, (photo-) electron diffraction, in general, is one of the most important tools to study adsorption geometries and surface reconstructions.

The latest addition to the imaging toolbox of ultrafast science is X-ray free electron lasers. They allow for unambiguous studies of crystalline condensed matter systems where the effects of intense ultrashort light pulses are probed using X-ray diffraction or absorption. Conventional concepts, which are valid at thermodynamic equilibrium, must often be modified in order to adequately describe time-dependent changes in the material properties. For example, a material may be described as a collection of quasi-thermal subsystems in thermal contact with each other or depicted as a function of the fully coherent dynamics of a complex network of excitations. X-ray lasers also allow us to study different subsystems in a crystalline condensed matter system via associated X-ray diffraction peaks. This gives access to the systems themselves but also correlations between them. In novel low-dimensional materials, topological protection and/or electronic correlations result in exotic charge, spin, or orbitally ordered ground states having new functionalities such as multiferroelectricity, high-temperature superconductivity, skyrmion magnetism, and Weyl semimetallicity.

The majority of papers in this special issue are concerned with the study of ultrafast phenomena in molecular and solid state systems. These studies have produced new scientific results and advances in theoretical and technological imaging methodologies, and promise to deliver exciting insights and developments in the future.

